# Transgenerational plasticity of reproduction depends on rate of warming across generations

**DOI:** 10.1111/eva.12386

**Published:** 2016-06-02

**Authors:** Jennifer M. Donelson, Marian Wong, David J. Booth, Philip L. Munday

**Affiliations:** ^1^ School of Life SciencesUniversity of TechnologySydneyBroadwayNSWAustralia; ^2^ ARC Centre of Excellence for Coral Reef StudiesJames Cook UniversityTownsvilleQLDAustralia; ^3^ Centre for Sustainable Ecosystems and SolutionsSchool of Biological SciencesUniversity of WollongongWollongongNSWAustralia

**Keywords:** acclimation, climate change, developmental plasticity, global warming, marine fish, transgenerational plasticity

## Abstract

Predicting the impacts of climate change to biological systems requires an understanding of the ability for species to acclimate to the projected environmental change through phenotypic plasticity. Determining the effects of higher temperatures on individual performance is made more complex by the potential for environmental conditions experienced in previous and current generations to independently affect phenotypic responses to high temperatures. We used a model coral reef fish (*Acanthochromis polyacanthus*) to investigate the influence of thermal conditions experienced by two generations on reproductive output and the quality of offspring produced by adults. We found that more gradual warming over two generations, +1.5°C in the first generation and then +3.0°C in the second generation, resulted in greater plasticity of reproductive attributes, compared to fish that experienced the same increase in one generation. Reproduction ceased at the projected future summer temperature (31.5°C) when fish experienced +3.0°C for two generations. Additionally, we found that transgenerational plasticity to +1.5°C induced full restoration of thermally affected reproductive and offspring attributes, which was not possible with developmental plasticity alone. Our results suggest that transgenerational effects differ depending on the absolute thermal change and in which life stage the thermal change is experienced.

## Introduction

Anthropogenic climate change is causing environmental conditions to shift from long‐term averages (Collins et al. 2013), and consequently represents a global threat to biodiversity (Thomas et al. 2004; Lovejoy and Hannah 2005). For conservation and management of ecosystems, a realistic understanding of the capacity for species to respond over years and generations is required (Hoffmann and Sgrò 2011). To persist within their current range, organisms could genetically adapt to the new environment or acclimate through phenotypic plasticity (Hoffmann and Sgrò 2011; Munday et al. 2013). Due to the pace of projected climate change and the potential constraints on rapid genetic adaptation, plasticity is expected to be especially important in enabling organisms to maintain their performance in the future (Gienapp et al. 2008; Merilä 2012). The ability of species to adjust to a changing environment will set the limits for the range and magnitude of biological impacts caused by climate change (Bradshaw and Holzapfel 2006; Bellard et al. 2012).

To ensure the persistence of populations in the future, successful reproduction is essential. Species have generally evolved to undertake reproductive activities within a narrow subset of temperatures compared to the thermal range in which they persist, due to the energetic cost associated (Van Der Kraak and Pankhurst 1997; Browne and Wanigasekera 2000; Visser et al. 2008). Consequently, environmental warming that has already occurred has resulted in shifts in the phenology of reproductive events for many species, allowing reproduction to still occur within the optimal thermal range (Parmesan and Yohe 2003; Dunn and Winkler 2010). Shifting reproductive timing is a risky strategy if a mismatch occurs between the timing of offspring production and their food availability (Cushing 1969; Visser and Holleman 2001; Edwards and Richardson 2004; Charmantier et al. 2008). Furthermore, some species will not shift their reproductive timing because they utilize environmental cues for reproduction that are not changing alongside warming (e.g. light; Hamner 1963; Both and Visser 2001; Bradshaw and Holzapfel 2010), and consequently may reproduce in suboptimal thermal conditions. This can result in a reduction in the quality and/or quantity of progeny produced, with potential negative implications for population persistence in the future (Giebelhausen and Lampert 2001; Donelson et al. 2010; Miller et al. 2015).

Environmental warming is expected to be especially problematic to ectotherms, due to their lack of internal thermal regulation. Cellular function is tightly linked to external thermal conditions and changes in physiological processes flow on to higher level performance, including reproduction (Pörtner 2002; Sunday et al. 2011). In fish, temperature experienced during juvenile development can affect later reproductive performance through its influence on sexual development (Davies et al. 1986; Pankhurst and Munday 2011), gender determination (Devlin and Nagahama 2002), and the timing of maturation (Davies et al. 1986; Pankhurst and Munday 2011). Temperature influences reproductive processes by promoting or inhibiting hormone synthesis, altering hormone structure, and modifying the action of hormones and enzymes within the hypothalamo–pituitary–gonadal (HPG) axis, resulting in affects to gametes and offspring produced (Pankhurst and Munday 2011). Short‐term experiments indicate that upper thermal thresholds for reproductive activity in many species of fish are only a few degrees above current‐day conditions (Van Der Kraak and Pankhurst 1997; Donelson et al. 2010; Pankhurst and King 2010). Some reproductive plasticity to moderate warming exists, but this plasticity appears to be limited when fish experience larger temperature changes, such as those predicted by the end of the century due to climate change (Donelson et al. 2014).

An important consideration when assessing the impacts of projected future temperatures on reproduction is that warming from climate change will occur over a number of generations for most species. Yet, relatively little is known about the capacity for plasticity of reproductive processes to warming over multiple generations (transgenerational plasticity: TGP). Various capacity for TGP has been observed between species and depending on the trait in question (Groeters and Dingle 1988; Shama and Wegner 2014; Walsh et al. 2014; Chakravarti et al. 2016). For example in the marine stickleback, Shama and Wegner (2014) observed positive TGP to offspring body size at day 30, negative effects to the number of eggs produced and no effect to egg size, while Chakravarti et al. (current special edition) found only partial compensation of fecundity with two generations in a marine polychaete. The coral‐reef damselfish, *Acanthochromis polyacanthus* (the model species utilized in this study) has been observed to produce beneficial TGP in response to warming, in other traits including aerobic physiology (Donelson et al. 2012a) and sex determination (Donelson and Munday 2015), but the capacity for TGP to influence reproduction is unknown. Full restoration of reproduction did not occur in *A. polyacanthus* with development at elevated temperatures, resulting in reductions in the amount and size of gametes produced compared with the previous generation (Donelson et al. 2014). However, full compensation of reproduction may be possible when several generations experience warmer conditions, allowing TGP to occur.

The rate of change experienced can influence the response to an environmental change such as warming. Generally, individuals tolerate higher maximum and minimum temperatures when conditions change rapidly, compared to more gradual changes (Terblanche et al. 2007; Chown et al. 2009; Peck et al. 2009). However, over the timescales at which plasticity can occur, gradual changes in temperature potentially allow a greater opportunity for plastic adjustment to higher temperature, whereas abrupt changes may cause greater costs due to time‐lags in response (Angilletta 2009). In a number of studies slower rates of temperature change corresponded to the observation of more beneficial plasticity (Kelty and Lee 1999; Allen et al. 2012; Westhus et al. 2013), but this is not always found (Schuler et al. 2011). In addition rapid environmental change, compared to gradual, may result in different outcomes at the population level due to selection of more stress‐tolerant genotypes that are more suited to rapid change (Bijlsma and Loeschcke 2005; Reusch and Wood 2007). One area of research that is ripe for investigation is how rates of change across generations might influence plastic outcomes. Gradual increase in temperature over generations may allow developmental plasticity to occur on top of TGP, possibly altering or amplifying the plasticity observed with only TGP (Shama and Wegner 2014). Additionally, environments that are continually changing may cause parents to bet hedge with offspring phenotype, due to their uncertainty about future conditions (Marshall et al. 2008; Simons 2014).

The present study explores the capacity for TGP to compensate for the negative effects of projected warming on reproductive capacity in a common reef damselfish, *Acanthochromis polyacanthus*. Specifically, we tested whether developing at +1.5 and +3.0°C for two generations enhanced reproductive ability above what was observed in the previous generation with only developmental plasticity. In addition, we included a step treatment where fish developed at +1.5°C in the first generation (F_1_) and then +3.0°C for the second generation (F_2_). This investigated the potential for additional developmental plasticity to +3°C warming on top of TGP expressed to +1.5°C. Finally, we tested the effects of transgenerational temperature treatment and reproductive temperature on the quantity and quality of offspring produced.

## Materials and methods

### Experimental design

The species used in this study was the coral reef damselfish *Acanthochromis polyacanthus*, a widespread Indo‐Pacific species (15°N–26°S and 116°E–169°E) that broods its young (Pankhurst et al. 1999). Fish were collected from the Palm Island region (18°37′S, 146°30′E) of the central Great Barrier Reef, which experiences a mean annual temperature range of 23.2–28.5°C (Australian Institute of Marine Science temperature loggers 1999–2008 at 6–8 m; http://data.aims.gov.au/). Average temperatures at the collection location have naturally fluctuated between 0.2–2.5°C in a single day, but on average vary only 0.45°C daily. Laboratory temperature treatments were maintained at ± 0.5°C of the desired temperature. In the 10 years leading up to the collection of F_0_ wild pairs, average summer temperatures for the collection region varied from 27.4–29.2°C. Average sea surface temperatures in the Great Barrier Reef, Australia, are predicted to increase up to 3°C by 2100 due to global warming (Lough 2007; Hobday and Lough 2011). Consequently, temperature treatments of +1.5°C or +3.0°C were chosen to reflect middle and end of the century projections.

**Figure 1:**
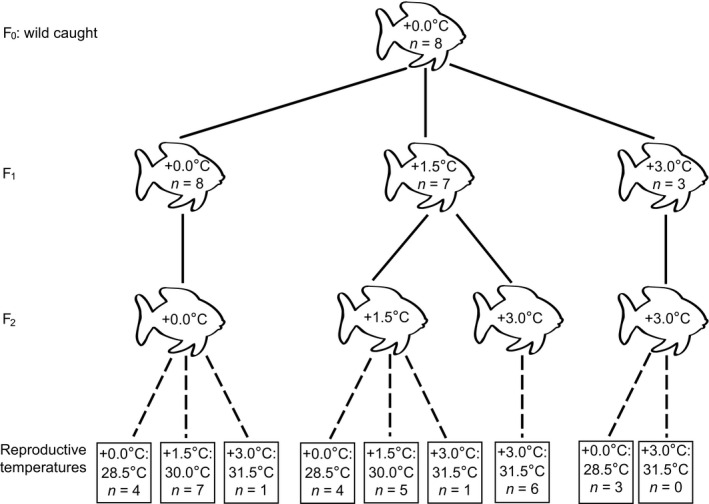
Experimental design where fish were reared in control (+0.0°C) or elevated thermal conditions (+1.5 and +3.0°C) from shortly after hatching for two generations.

The experimental design involved two factors: Generation (F_1_, F_2_) and Rearing temperature (present‐day, +1.5°C, +3.0°C). *A. polyacanthus* were reared for two generations at present‐day and elevated temperatures. At the start of the experiment, eight adult breeding pairs (F_0_) were collected from the Palm Island region and maintained at present‐day summer temperatures for 4 months (Donelson et al. 2010). The first clutch of offspring (F_1_) produced by these breeding pairs were divided among the three temperature treatments and reared to maturity (Fig. 1). During summer when breeding occurs and offspring develop, the average temperatures were: present‐day = 28.5°C, +1.5°C = 30.0°C and +3.0°C = 31.5°C. All the treatments followed the natural seasonal cycle for the collection location; therefore, respective winter temperatures were lower in each treatment (see Donelson et al. 2011). The dark–light cycle was also matched weekly to seasonal changes in day length for the collection location, summer was approximately 13L:11D and winter was 11L:13D. Sibling fish were kept in groups of 6 in 40 L aquaria for 1 year after hatching, at which time density was reduced to pairs by the experimenter. Mortality with in the sibling groups was low, with >90% survival in all treatments. At 1.5 years fish were reorganized using individuals from the same temperature treatment into nonsibling pairs for breeding at 2 years when maturity was reached (see Donelson et al. 2014 for more details). Nonsibling pairs were composed of an even number of individuals from each parental line. Fish were fed a stage‐specific commercial fish food as described by Donelson et al. (2011).

Clutches of offspring (F_2_) from the F_1_ breeding pairs were divided into the different temperatures at hatching and reared as described above for the F_1_ generation (Fig. 1). At maturity, F_2_ individuals were again outcrossed to form new breeding pairs as in the previous generation. Two months prior to summer, pairs from the main multigenerational lineages were further divided across the three treatments to investigate the independent and interacting effects of multigenerational temperature and reproductive temperature exposure (Fig. 1). There was close to 100% survival in all temperature treatments in both the F_1_ and F_2_ generations.

### Reproduction and offspring characteristics

During the austral summer 2011–2012 nesting sites of F_2_ breeding pairs were checked daily at 09:00 for the presence of eggs. When a clutch was discovered an underwater photograph was taken to estimate the number of eggs laid and a sample of 10 eggs from random locations within the clutch was taken to determine egg size (to nearest 0.01 mm, see Donelson et al. 2011 for methods). Following the observation of a clutch, tanks were checked again daily at 11:00 for the presence of hatched offspring. Directly after hatching, a sample of 20 offspring were removed and euthanized to subsequently determine offspring characteristics with image analysis (Image J; standard length to nearest 0.01 mm: SL, weight to nearest mg: W and yolk area to nearest 0.01 mm: YA). Fulton's K condition was calculated as (W/SL^3^) × 100. To determine mean egg size and offspring attributes at hatching (SL, W, Fulton's K condition and YA) only the first clutch produced by each pair were used. To determine mean number of eggs all clutches produced during the breeding season were used. Total progeny produced in each treatment was calculated as the sum of all the eggs produced by pairs throughout the breeding season. The timing of breeding within each treatment was calculated as the duration of breeding across all months of the summer season. Specifically, it was the length of time from the first to the last clutch in each treatment. The average time that breeding commenced was calculated as the mean week for the first clutch of all pairs that bred within a treatment.

### Analyses

As data were normally distributed, reproductive timing and duration, clutch characteristics and offspring attributes were compared among treatments with general linear models (GLM). Each combination of grandparent temperature, parent temperature, and reproductive temperature was considered a treatment (Fig. 1). Factors included within the GLM were the temperature treatment (fixed), maternal weight (covariate), and pair ID (random). Groups with only one pair reproducing were excluded from the GLMs as there was no replication at the pair level (+0.0 at 31.5°C and +1.5 at 31.5°C). Where significant differences were found (*P* < 0.05), post hoc testing was completed with Student's T pairwise comparisons. The proportion of pairs that reproduced per combination treatment was explored with individual 2 × 2 chi squared analysis where the expected distribution was the control (+0.0 at 28.5°C). All statistical analyses were completed with JMP^®^, Version 11, SAS Institute Inc., Marlow, Buckinghamshire, UK. Assumptions of statistical models were tested with q‐q plots and Levene's tests.

## Results

### Breeding characteristics

**Figure 2:**
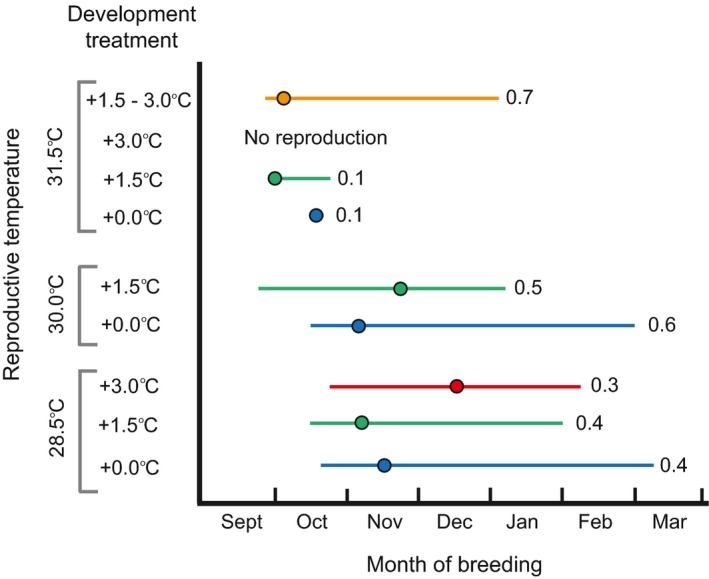
Seasonal duration of breeding in developmental treatments depending on reproductive temperature (lines). Circles denote the mean month of breeding commenced and values adjacent to lines indicate the proportion of pairs that contributed to reproduction.

Reproduction in F_2_ pairs occurred in all combinations of developmental treatment and reproductive temperature, except for +3.0°C fish housed at 31.5°C, where no pairs reproduced during the entire summer (Fig. 2; +3.0°C at 31.5°C vs +0.0°C at 28.5°C: *X* ^2 ^= 6.29, df = 1, *P* = 0.01). A reduction in the number of pairs that reproduced was observed for both +0.0°C and +1.5°C treatments at 31.5°C. In both cases only one pair reproduced representing 10% of the possible pairs in each treatment, however this was not statistically significant from the control proportion of 0.4 (*X* ^2 ^= 2.76, df = 1, *P* = 0.10). The percent of pairs breeding was similar between all other treatments (*P* > 0.05).

Breeding commenced during September and October in all combinations of developmental treatment and reproductive temperature, but the average month that pairs commenced reproduction was affected by the treatment and temperature combination (Fig. 2; *F*_5,23 _= 2.88, *P* = 0.04). This effect was driven by fish whose parents experienced +1.5°C (F_1_ generation) and they developed at +3.0°C (step treatment +1.5 – +3.0°C) breeding earlier on average (Fig. 2; *P* < 0.05 for +0.0°C at both 28.5°C and 30.0°C, +1.5°C at 28.5°C, and +3.0°C at 28.5°C). In present‐day control (28.5°C) and elevated 30°C conditions, reproduction tended to occur from October to early March (Fig. 2). As reproductive temperature increased to 31.5°C, reproduction occurred only in September to October for +0.0 and +1.5°C fish. Contrastingly, fish that experienced +1.5°C in the F_1_ generation and +3.0°C in the F_2_ generation extended their breeding season to occur for a similar length (September to January) as pairs at cooler reproductive temperatures (*F*_5,23 _= 1.21, *P* = 0.33).

### Clutch characteristics

**Figure 3:**
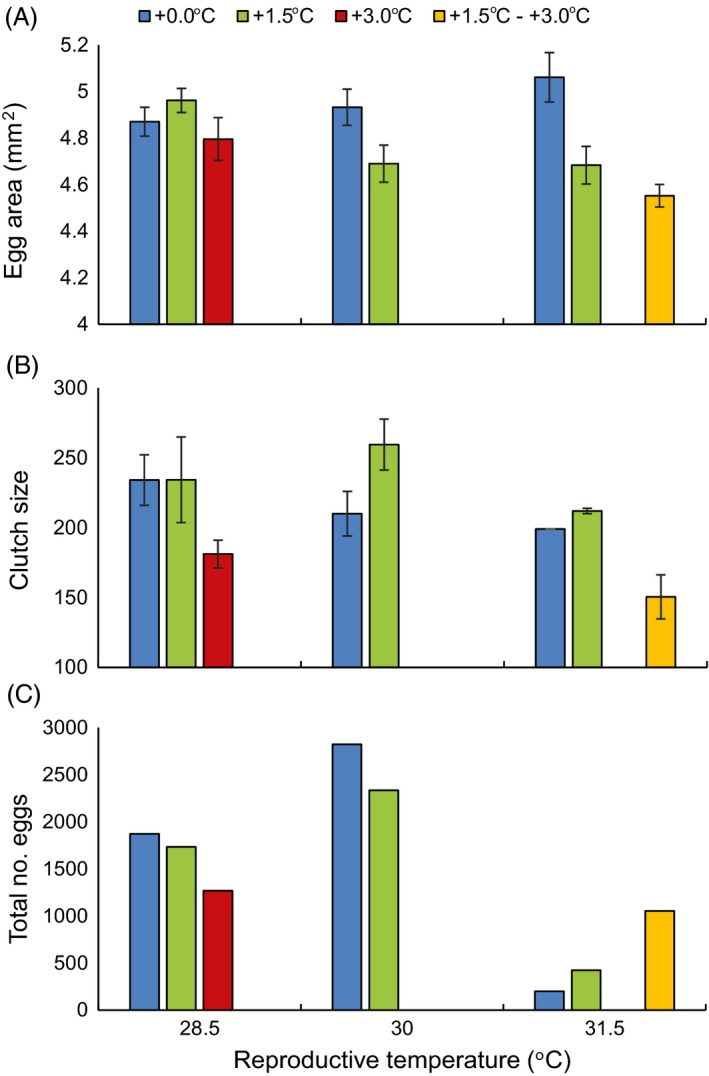
Mean (±SE) egg area (A), clutch size (B), and total number of eggs produced in the summer breeding season (C) of *Acanthochromis polyacanthus* pairs depending on multigenerational temperature treatment and reproductive temperature.

The average area of eggs differed from 4.5 to 5.1 mm^2^ between combination treatments (Fig. 3A), but there was no relationship with breeding temperature (*F*_7,17 _= 0.48, *P* = 0.84; Table 1), possibly due to high variability between pairs within treatments (Pair ID: *z* = 2.77, *P* < 0.05). In contrast, the average number of eggs produced per clutch did differ depending on combination treatment (Fig. 3B; *F*_5,16 _= 3.07, *P* = 0.04; Table 1). For fish from developmental treatment +0.0 and +1.5°C, the number of eggs per clutch was maintained across breeding temperatures (*P* > 0.05). Fish reared at +3.0°C for two generations tended to produce 30% smaller clutches at 28.5°C, however this was not significant (*P* > 0.05). Egg clutches of fish elevated to 3.0°C over 2 generations (step treatment +1.5 – +3.0°C) were 64% smaller than control pairs (+0.0 at 28.5°C, *P* = 0.02). The total number of eggs produced per season peaked at the breeding temperature of 30.0°C in both the +0.0 and +1.5°C treatments (Fig. 3C). This trend was driven by slightly more pairs reproducing and producing more clutches. At the control temperature of 28.5°C, the difference in total progeny produced between +3.0°C and +0.0°C was the same reduction as observed in average clutch size (0.7). While fish from the step treatment (+1.5 – +3.0°C) produced only half the offspring compared to control +0.0 at 28.5°C, they produced approximately 3.5 times the number of eggs compared to either the +0.0 or +1.5°C fish at the same reproductive temperature (31.5°C).

**Table 1:** Statistical results of generalized linear models for testing differences in egg area and clutch size between temperature treatments

	Effect type	Estimate ± SE	Statistic	*P* value
Egg area				
Treatment	Fixed		*F*_7,17 _= 0.481	0.84
Maternal weight	Fixed		*F*_1,17 _= 0.068	0.80
Pair	Random	0.177 ± 0.064	*Z *=* *2.77	*<0.05*
Residual		0.089 ± 0.008		
Clutch size				
Treatment	Fixed		*F*_5,16 _= 3.072	*0.04*
Maternal weight	Fixed		*F*_1,16 _= 2.448	0.14
Pair	Random	757.75 ± 851.92	*Z *=* *0.889	>0.05
Residual		2248.53 ± 705.18		

Italics denotes significant *p* values.

### Offspring characteristics

**Figure 4:**
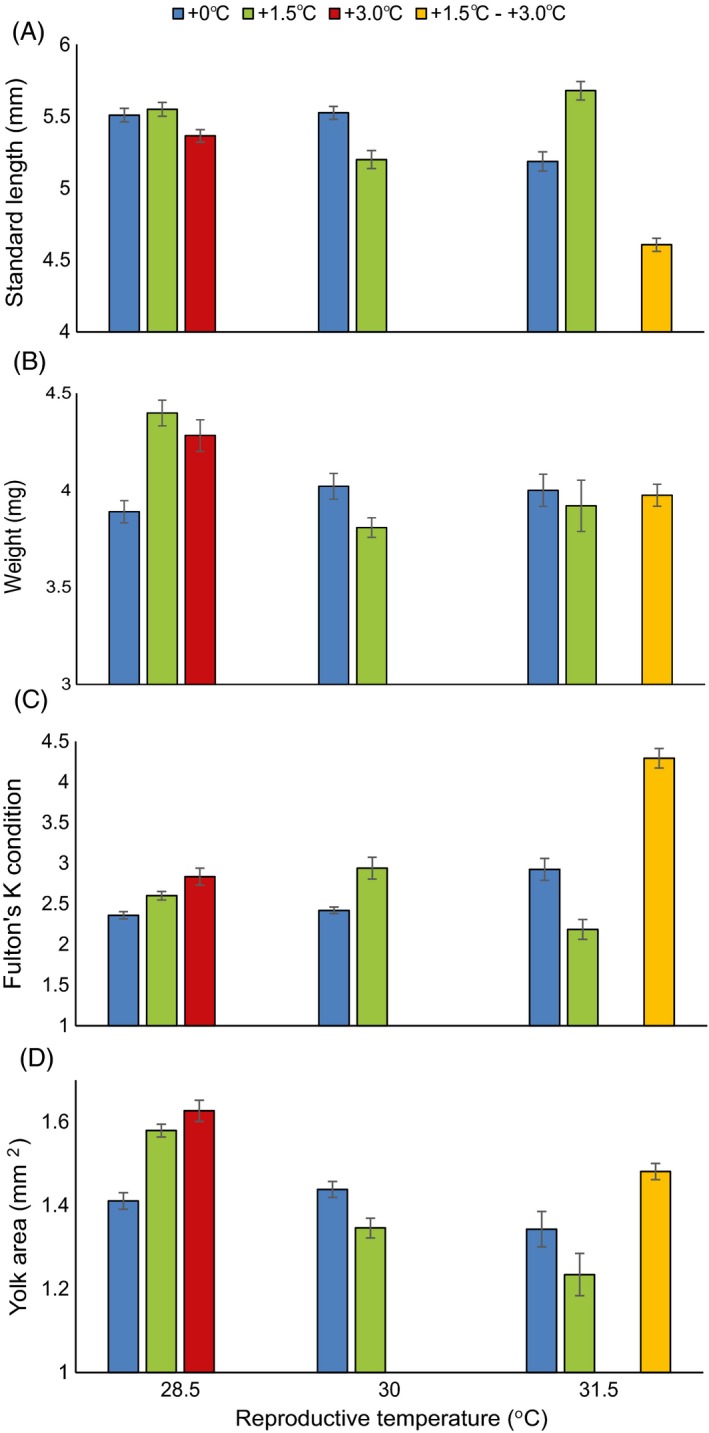
Mean standard length (A), weight (B), Fulton's K condition index (C), and yolk area (D) of *Acanthochromis polyacanthus* offspring from pairs depending on multigenerational temperature treatment and reproductive temperature. Values are mean (±SE).

The physical attributes of offspring were influenced by the combination treatment of parents, but the pattern differed among the attributes measured (Fig. 4; Table 2). Offspring of pairs from both the +1.5 and +3.0°C treatment for two generations that reproduced at 28.5°C exhibited an increase in the weight and the amount of provisioning compared with +0.0°C at 28.5°C (Fig. 4B,D), however this trend was not significant (W: *F*_5,19 _= 1.14, *P* = 0.37; YA: *F*_5,19 _= 2.14, *P* = 0.10), possibly due to the high variability between pairs within treatments (W: *z* = 3.01, *P* < 0.05; YA: *z* = 2.68, *P* < 0.05) and the effect of maternal size on offspring weight (*F*_1,19 _= 5.45, *P* = 0.03). Offspring produced by pairs in the step treatment (+1.5 – +3.0°C) were significantly shorter than all other treatments, except 1.5°C at 30°C, by >0.6 mm (Fig. 4A; SL: *F*_5,19 _= 3.36, *P* = 0.03). While these offspring were shorter, their weight was similar to other treatments, causing them to have a significantly higher physical condition index than all other groups (Fig. 4C; Fulton's K condition: *F*_5,19 _= 5.86, *P* = 0.002) after controlling for pair variability (SL: *z* = 3.03, *P* < 0.05; Fulton's K condition: *z* = 2.96, *P* < 0.05).

**Table 2:** Statistical results of generalized linear models for testing differences in offspring characteristics between temperature treatments

	Effect type	Estimate ± SE	Statistic	*P* value
Standard length				
Treatment	Fixed		*F*_5,19 _= 3.363	*0.02*
Maternal weight	Fixed		*F*_1,19 _= 0.200	0.66
Pair	Random	0.202 ± 0.067	*Z* = 3.030	*<0.05*
Residual		0.068 ± 0.004		
Weight				
Treatment	Fixed		*F*_5,19 _= 1.139	0.37
Maternal weight	Fixed		*F*_1,19 _= 5.449	*0.03*
Pair	Random	0.266 ± 0.088	*Z* = 3.015	*>0.05*
Residual		0.113 ± 0.007		
Fulton's K				
Treatment	Fixed		*F*_5,19 _= 5.856	*0.002*
Maternal weight	Fixed		*F*_1,19 _= 1.306	0.26
Pair	Random	0.494 ± 0.167	*Z* = 2.961	*<0.05*
Residual		0.026 ± 0.026		
Yolk area				
Treatment	Fixed		*F*_5,19 _= 2.145	0.10
Maternal weight	Fixed		*F*_1,19 _= 0.560	0.46
Pair	Random	0.012 ± 0.004	*Z* = 2.684	*>0.05*
Residual		0.033 ± 0.002		

## Discussion

Conservation of biological systems in the face of future climate change requires an understanding of the ability for species and populations to adjust to rising temperatures over relevant time scales, however this has been rarely done to date. Experimental investigations of species’ cross‐generational responses to projected change represents one of the best methods we have for estimating the likely biological impacts (Munday et al. 2013; Munday 2014 for reviews). Our study highlights that the generational rate of warming applied in experiments can alter the phenotypic results. We found that relatively gradual warming, with a +1.5°C increase per generation, over two generations, resulted in enhanced reproduction compared to a + 3.0°C increase in the F_1_ generation. Fish that developed for two generations at +3.0°C were unable to reproduce at all in the expected future summer conditions (31.5°C). Additionally, we found evidence for TGP, greater than what was possible with developmental plasticity alone, in egg and offspring attributes for fish maintained at +1.5°C for two generations. However, this TGP did not provide reproductive benefits at temperatures greater than they had experienced.

Slower rates of increase may produce enhanced plasticity as they allow further developmental plasticity to occur on top of TGP. This was evidenced in the current study by the enhanced number of pairs reproducing, and consequently progeny produced, in the +1.5 – +3.0°C step treatment compared to the +1.5°C treatment group at 31.5°C, since the difference between these two treatments is only development of the step treatment during the F_2_ generation at +3.0°C. Previous research has also found that the environment experienced by marine fish at both the present and previous generation can independently influence the quality and success of offspring produced, although not always in the same direction (Shama and Wegner 2014). Here, we found that a step‐wise increase in temperature over two generations provided the most benefits to reproduction compared with simply exposing fish to the same higher temperatures over the same timeframe. It is well‐established that the rate of warming experienced, relative to natural conditions, will influence the thermal tolerance of individuals, whether they mount a stress response (Feder and Hofmann 1999; Hofmann and Todgham 2010). Our finding, that a more gradual increase over generations promoted plasticity, matches with previous findings that slower environmental changes allows time for within generation plasticity to occur (Kelty and Lee 1999; Angilletta 2009; Allen et al. 2012; Westhus et al. 2013). While many reproductive attributes were improved in the +1.5 – +3.0°C step treatment, such as offspring physical condition, there were still limitations to plasticity with lower numbers of smaller progeny produced compared to control +0.0°C. Further improvements in reproductive capacity might be expected with more gradual warming over greater generations.

Reproduction ceased in fish maintained at +3.0°C for two generations, and held at 31.5°C during summer. However, fish from the same developmental treatment were still able to reproduce at 28.5°C, indicating there was a limitation to reproducing at the warm temperature rather than permanent reproductive disruption, such as abnormal gonadal development, caused by the high temperature (Van Der Kraak and Pankhurst 1997). Nevertheless, the number of eggs produced by +3.0 pairs at 28.5°C was lower, compared to both +0.0 and +1.5°C, indicating that development at +3.0°C for multiple generations came at some cost. For example, development in warmer conditions may still have resulted in some alteration to gonadal structure or function (Van Der Kraak and Pankhurst 1997). Elevated water temperature causes a greater rate of masculinization in *A. polyacanthus*, possible having implications for males that otherwise would have developed as females in normal thermal conditions (Donelson and Munday 2015). Alternatively, breeding failure for fish maintained multi‐generationally at +3.0°C could indicate that they have opted to delay breeding to a later year (Sandström et al. 1995).

Transgenerational plasticity, above what was observed only with developmental plasticity, allowed compensation of all egg and offspring attributes by +1.5°C fish that reproduced at 30.0°C. Egg and offspring attributes in this group match +0.0°C control fish at 28.5°C. In the previous generation the mean egg size as well as the length and weight of offspring was unable to be restored with development only (i.e. partial compensation, Donelson et al. 2014). While TGP enhanced reproduction and offspring attributes of +1.5°C fish breeding at 30°C, there was no benefits observed at temperatures warmer than what they developed in (i.e. 31.5°C). This suggests that beneficial phenotypic alterations to the endocrine system require experience of the thermal environment, in contrast with research on the same fish earlier in life where TGP at +1.5°C enhanced aerobic physiology across all testing temperatures, including at 31.5°C (Donelson et al. 2012a).

The phenotypic differences observed in this study could also be influenced by selection within the treatments, because not all breeding pairs reproduced. Some selection was observed in the previous generation within the +3.0°C treatment (Donelson et al. 2012a). Consequently, the poorer breeding outcomes for the +3.0°C could be due to at least in part to this selection in the previous generation. While, the improved reproduction of +1.5 – 3.0°C step treatment could also be a result of selection, this is less likely than TGP, and unlikely to be the primary mechanism. There was no evidence for particular parental lines disproportionately composing the pairs that reproduced in the +1.5 – 3.0°C step treatment, compared to those that did not reproduce. Additionally, there was no evidence that particular parental lines disproportionately composed reproducing pairs in the +1.5 – 3.0°C step treatment compared to those that reproduced in the +1.5°C treatment at 28.5 or 30°C.

Shifts in the phenology of reproductive migrations and breeding have been widely documented in relation to ocean warming that has already occurred (Poloczanska et al. 2013; Asch 2015) and are a commonly predicted response to further warming (Munday et al. 2009). In previous experiments with *A. polyacanthus* we have not observed any evidence for shifting breeding timing forward with acute or developmental exposure at higher temperatures (Donelson et al. 2010, 2014). By contrast, in the current study the fish in the multigenerational thermal‐step treatment (+1.5 – +3.0°C) commenced breeding earlier on average. Shifting reproduction will likely reduce costs to adults as reproduction would occur prior to the warmest parts of summer, where basal metabolic costs are lower (Donelson et al. 2012a). However, whether this timing shift is beneficial or negative in the longer term would ultimately depend on any phenological shifts that occur in the prey for the offspring (Visser and Holleman 2001; Edwards and Richardson 2004; Charmantier et al. 2008).

Our results on the acute effects of warming to reproductive capacity differed slightly from previous work on wild pairs (Donelson et al. 2010, 2012b). Formerly more drastic effects of 1.5°C warming (30.0°C) were seen, with reductions in the percentage of pairs reproducing and the quality of progeny produced (Donelson et al. 2010, 2012b). Difference between studies are possibly due to the age, size, and experience of fish, with wild breeding pairs used in previous experiments being larger, older and unlikely to be first time breeders. Differences could also be due to selection that has occurred over generations in the lab potentially skewing the response of individuals to perform better at 30°C (Hoffmann et al. 2001).

It is likely that TGP will be an essential pathway for restoration of thermally sensitive processes in many species as the climate continues to change. A growing number of experiments are improving our understanding of the potential for phenotypic responses across generations, but when obtaining estimates of TGP we need to be cautious with how we conduct the experiments and consequently extrapolate the findings to conservation objectives. This study indicates that immediately increasing the temperature to future projected levels may not fully elucidate the true capacity for plasticity that will occur over multiple generations in the next 50–100 years as it does not allow interactions between developmental and transgenerational pathways. This poses a risk that our current comprehension of TGP may incorrectly overestimate the impacts to species and populations. Designing species specific relevant experiments will be essential to ensure the most accurate estimates of future plastic capacity on which to make management decisions.

## Data archiving statement

Data for this study are available the Dryad Digital Repository: http://dx.doi.org/10.5061/dryad.qg15c.
